# ChromID^®^ CARBA Agar Fails to Detect Carbapenem-Resistant *Enterobacteriaceae* With Slightly Reduced Susceptibility to Carbapenems

**DOI:** 10.3389/fmicb.2020.01678

**Published:** 2020-08-11

**Authors:** Natalie Pauly, Jens A. Hammerl, Mirjam Grobbel, Bernd-Alois Tenhagen, Annemarie Käsbohrer, Sandra Bisenius, Jannika Fuchs, Sabine Horlacher, Holger Lingstädt, Ute Mauermann, Silke Mitro, Margit Müller, Stefan Rohrmann, Arthur P. Schiffmann, Birgit Stührenberg, Pia Zimmermann, Stefan Schwarz, Diana Meemken, Alexandra Irrgang

**Affiliations:** ^1^German Federal Institute for Risk Assessment, Berlin, Germany; ^2^Unit for Veterinary Public Health and Epidemiology, University of Veterinary Medicine, Vienna, Austria; ^3^Institute for Fish and Fishery Products (LAVES), Cuxhaven, Germany; ^4^Chemical and Veterinary Investigation Office, Karlsruhe, Germany; ^5^Chemical and Veterinary Investigation Office, Fellbach, Germany; ^6^State Office for Consumer Protection Saxony-Anhalt, Stendal, Germany; ^7^Food and Veterinary Institute, Oldenburg, Germany; ^8^State Investigation Institute for Health and Veterinary Services, Chemnitz, Germany; ^9^Chemical and Veterinary Investigation Office Rhein-Ruhr-Wupper, Krefeld, Germany; ^10^Chemical and Veterinary Investigation Office, Arnsberg, Germany; ^11^Hessian State Laboratory, Gießen, Germany; ^12^Chemical and Veterinary Investigation Office, Detmold, Germany; ^13^Bavarian Health and Food Safety Authority, Oberschleißheim, Germany; ^14^Institute of Microbiology and Epizootics, Freie Universität Berlin, Berlin, Germany; ^15^Institute of Food Safety and Food Hygiene, Freie Universität Berlin, Berlin, Germany

**Keywords:** carbapenemase, isolation, media, specificity, sensitivity

## Abstract

After first detections of carbapenemase-producing Enterobacteriaceae (CPE) in animals, the European Union Reference Laboratory for Antimicrobial Resistance has provided a protocol for the isolation of carbapenemase-producing *Escherichia* (*E.*) *coli* from cecum content and meat. Up to now, only few isolates were recovered using this procedure. In our experience, the choice of the selective agar is important for the efficacy of the method. Currently, the use of the prevailing method fails to detect CPE that exhibit a low resistance against carbapenems. Thus, this study aims to evaluate the suitability of selective media with antibiotic supplements and commercial ChromID^®^ CARBA agar for a reliable CPE detection. For comparative investigations, detection of freeze-dried carbapenemase-resistant bacteria was studied on different batches of the ChromID^®^ CARBA agar as well as on MacConkey agar supplemented with 1 mg/L cefotaxime and 0.125 mg/L meropenem (McC+CTX+MEM). The suitability of the different media was assessed within a time of 25 weeks, starting at least six weeks before expiration of the media. Carbapenem-resistant isolates exhibiting a serine-based hydrolytic resistance mechanism (e.g., *bla*_KPC_ genes) were consistently detected over 25 weeks on the different media. In contrast, carbapenemase producers with only slightly reduced susceptibility and exhibiting a zinc-catalyzed activity (e.g., *bla*_VIM_, *bla*_NDM_, and *bla*_IMP_) could only be cultivated on long-time expired ChromID^®^ CARBA, but within the whole test period on McC+CTX+MEM. Thus, ChromID^®^ CARBA agar appears to be not suitable for the detection of CPE with slightly increased minimum inhibitory concentrations (MIC) against carbapenems, which have been detected in German livestock and thus, are of main interest in the national monitoring programs. Our data are in concordance with the results of eleven state laboratories that had participated in this study with their ChromID^®^ CARBA batches routinely used for the German CPE monitoring. Based on the determined CPE detection rate, we recommend the use of McC+CTX+MEM for monitoring purposes. This study indicates that the use of ChromID^®^ CARBA agar might lead to an underestimation of the current CPE occurrence in food and livestock samples.

## Introduction

Carbapenems are important antimicrobial substances with broad activity against almost all β-lactams. They are essential to treat severe human infections with multidrug-resistant Gram-negative bacteria. Due to their high impact in human medicine, dissemination of carbapenem-resistant bacteria should be avoided (Sharland et al., 2018; European Medicines Agency [EMA], 2019). Actually, more than 2,000 variants of carbapenemase resistance genes have been described that were allocated to the β-lactamases of Ambler classes A, B, and D (Codjoe and Donkor, 2017; Grundmann et al., 2017). *Klebsiella pneumoniae* carbapenemases (KPC) and defined OXA enzymes are common Ambler class A and D carbapenemases, respectively, with a serine residue in their active sites (Codjoe and Donkor, 2017). New Delhi metallo-β-lactamases (NDM) and Verona integron-encoded metallo-β-lactamases (VIM) are predominant Ambler class B enzymes. Their resistance mechanism depends on the interaction of the carbapenems with zinc ions in the active site of the enzymes (Codjoe and Donkor, 2017). Carbapenemase genes are often located on mobile genetic elements (i.e., transposons/integrons, plasmids, phages), which support horizontal transmission of the genes between different bacteria (Carattoli, 2009; Miriagou et al., 2010; Tato et al., 2010). The genes are often co-located with genes mediating resistance to other antimicrobial classes. This leads to an important reduction of therapeutic options in human medicine (Woodford et al., 2014). In addition, various chromosomal mechanisms are also involved in resistance development against carbapenems, i.e., changes in membrane permeability due to the loss of specific outer membrane porins (Miriagou et al., 2010).

For a long time, carbapenemase-producing Enterobacteriaceae (CPE) were almost exclusively associated with human medicine. However, the number of reported isolates from wildlife, companion animals, livestock, and food increases worldwide (Köck et al., 2018). To get deeper insights into the dynamics of occurring CPE, the European Union (EU) set up a carbapenem-resistance monitoring for *Escherichia coli* isolates from food products and livestock (Schrijver et al., 2018). Aside from clinical settings, the carbapenemase enzymes OXA, VIM, NDM, and KPC possess the highest impact in wildlife, pets, and the food chain (Grundmann et al., 2017; Köck et al., 2018). In Germany, France, Italy, Spain, and the Netherlands, reports have been published about carbapenem-resistant Enterobacteriaceae (CRE) in companion animals (i.e., dogs, cats, horses) (Stolle et al., 2013; Schmiedel et al., 2014; Gonzalez-Torralba et al., 2016; Melo et al., 2017; Vittecoq et al., 2017; Pulss et al., 2018), seafood (i.e., shrimps, blue mussels, cockles) (Roschanski et al., 2016; Ceccarelli et al., 2017), wild animals (i.e., yellow-legged gulls, black kite) (Fischer et al., 2013b; Vergara et al., 2017; Vittecoq et al., 2017), and food of livestock animals (i.e., beef, chicken meat, pork) (Schwaiger et al., 2008; Leverstein-van Hall et al., 2011; Poirel et al., 2012; Zurfluh et al., 2016; Ceccarelli et al., 2017; Fischer et al., 2017; Pulss et al., 2017; Randall et al., 2017; Roschanski et al., 2017, 2018). In 2011, VIM-1-producing Enterobacteriaceae (*Salmonella* Infantis and *E. coli*) were found in several chicken and pig farms in Germany (Borowiak et al., 2017; Falgenhauer et al., 2017; Fischer et al., 2017; Irrgang et al., 2017, 2019; Roschanski et al., 2018). The European Union Reference Laboratory for Antimicrobial Resistance (EURL-AR) together with national experts on antimicrobial resistances developed a culture-dependent procedure for the isolation of carbapenemase-producing *E. coli* from meat and cecum content samples^1^. As no validated method for isolates from food exists, this method is currently used in the annual monitoring on carbapenemase-producing *E. coli* from livestock and food in Germany and other European member states. In Germany, we recognized on several occasions within the monitoring and during additional studies that the method failed to detect CPE that were identified in the same sample using a different method. One example was an isolate in 2017, which was detected in the monitoring on ESBL/AmpC β-lactamases but could not be detected within the German CPE monitoring. It was a VIM-1-producing *E. coli* from the cecum content of a fattening pig at slaughter that exhibited minimum inhibitory concentrations (MIC) ranging around the current cutoff values for the tested carbapenems. These cutoff values are considerably lower than the MIC of CPE of human clinical origin (European Food Safety Authority [EFSA], and European Centre for Disease Prevention and Control [ECDC],, 2017, 2019). Thus, there was the necessity to improve the isolation method for CPE with low carbapenem MIC values from food and livestock samples.

Due to the prevailing deficit in reliable detection of CPE exhibiting low MIC values, this study aims to determine the sensitivity of the selective cultivation after enrichment of the samples for the laboratory routine in detecting CPE. Thus, the suitability of different selective media for the recovery of CPE was assessed. Beside the frequently used ChromID^®^ CARBA agar (bioMérieux, Nürtingen, Germany) (Nordmann et al., 2012; Girlich et al., 2013; Papadimitriou-Olivgeris et al., 2014), which is also predominantly used by the German federal state laboratories, we also determined the sensitivity of MacConkey agar supplemented with cefotaxime and meropenem over a time interval of 25 weeks.

## Materials and Equipment

### Bacterial Strains

Five carbapenem-resistant isolates were used for the evaluation of the different selective media (Table 1). We chose three CPE (*E. coli*: CP-3 and CP-9; *Salmonella* Corvallis: CP-5), which had previously been recovered from German food and livestock samples (Fischer et al., 2013b; Irrgang et al., 2017), one carbapenemase-producing *Vibrio parahaemolyticus* (CP-8) from seafood and one carbapenem-resistant *K. pneumoniae* isolate from a pig slaughterhouse that lacks carbapenemase production (CR-1). As references for quality assurance concerning the selectivity of the media, the ESBL-producing *E. coli* isolate AR-1 (Wilkinson et al., 2012) and *E. coli* ATCC 25922 were used.

**TABLE 1:** Strains and species used in this study with their resistance determinants (threefold determined MIC values, variations are indicated *with a “/” between the values*).

**Isolate**	**Bacterium**	**Origin**	**Carbapenemase genes**	**Carbapenemase production**	**ERP (mg/L)**	**IMI (mg/L)**	**MEM (mg/L)**	**References**
ATCC 25922	*E. coli*	DSMZ	None	−	≤0.015	0.25	≤0.03	DSMZ
AR-1	*E. coli*	Bovine cecum	None	−	0.25	0.5	0.6	This work
CP-3	*E. coli*	Colon content of a slaughter pig	*bla*_VIM–__1_	+	0.12	4	1	Irrgang et al., 2017
CP-5	*Salmonella* Corvallis	Wild bird	*bla*_NDM–__1_	+	>2	4	8	Fischer et al., 2013b
CP-8	*V. parahaemolyticus*	Seafood	*bla*_NDM–__1_	+	0.25	1/2/4	0.25	This work
CP-9	*E. coli*	Human	*bla*_KPC–__2_	+	>2	4	8	This work
CR-1	*K. pneumoniae*	Porcine cecum	None	−	2	0.5/1	0.5	This work

*ERP, ertapenem; IMI, imipenem; MEM, meropenem. DSMZ, German collection of microorganisms and cell cultures. Moreover CP, carbapenemase-producing; CR, carbapenem-resistant; and AR, antibiotic resistant.*

Further information on the bacterial isolates used in this study is summarized in Table 1. Detailed information on the MIC values of the isolates for 14 antimicrobials (ampicillin, cefotaxime, ceftazidime, chloramphenicol, ciprofloxacin, colistin, florfenicol, gentamicin, kanamycin, nalidixic acid, streptomycin, sulfamethoxazole, tetracycline, and trimethoprim) is provided in the Supplementary Table S1.

### Media and Supplements

If not stated otherwise, all bacteria were cultivated in lysogeny broth (LB) at 37°C for 16–20 h under shaking conditions (120–160 rpm). Solid media agars containing 0.8% (w/v) Bacto agar (Oxoid, Dassel, Germany) were used. If necessary, antimicrobial substances were supplemented to the media as indicated. All media (listed in Table 2) of this study were stored at 4–6°C in a refrigerator and only exposed to light during processing of the experiments.

**TABLE 2:** List of media, reagents, materials, and equipment.

**Material**	**Produced by**	**Further information**	**Country**
Lysogeny broth	*In-house*	Yeast extract 5 g/L; peptone from casein 10 g/L; NaCl 10 g/L	
Lysogeny agar	*In-house*	Yeast extract 5 g/L; peptone from casein 10 g/L; NaCl 10 g/L; agar 12 g/L	
Bacto agar	Oxoid		Dassel, Germany
Lyophilization medium	*In-house*	Glutamic acid 10.0 g/L; NaOH (8%) 30.0 mL/L; Bacto tryptone 25.0 g/L; sucrose 50.0 g/L; thiourea (CH_4_N_2_S) 2.0 g/L	
MacConkey agar	*In-house*	Peptone from casein 17 g/L; peptone from meat 3 g/L; NaCl 5 g/L; lactose 10 g/L; bile salt mixture 1.5 g/L, neutral red 0.03 g/L; crystal violet 0.001 g/L, agar 13.5 g/L	
ChromID^®^ CARBA	bioMérieux		Nürtingen, Germany
Cefotaxime	Sigma-Aldrich		Taufkirchen, Germany
Meropenem	Sigma-Aldrich		Taufkirchen, Germany
Plates EUVSEC and EUVSEC2	Thermo Fisher Scientific		Schwerte, Germany
Epsilon 2-10D LSC lyophilization instrument	Christ		Osterode am Harz, Germany
Sensititre AIM^TM^ Automated Inoculation Delivery System	TREK Diagnostic		East Grinstead, United Kingdom
Sensititre^TM^ SWIN^TM^ Software System 3.3	Thermo Fisher Scientific		Schwerte, Germany

## Methods

### Antimicrobial Susceptibility Testing

The susceptibility to antimicrobials of the isolates was determined by broth microdilution using defined antimicrobial substances and concentrations from the harmonized EU panel [plates EUVSEC and EUVSEC2; TREK Diagnostic Systems (Thermo Fisher Scientific, Schwerte, Germany)]. Resistance testing was conducted according to the EN ISO20776-1:2006 (European Food Safety Authority [EFSA], and European Centre for Disease Prevention and Control [ECDC],, 2018), and MIC values were interpreted based on EUCAST definitions^2^ using epidemiological cutoff values. The testing was repeated three times (Supplementary Table S1). As quality control strain, *E. coli* ATCC 25922, was used.

### Preparation of Freeze-Dried Bacteria

To ensure that equal amounts of bacterial cells were used throughout the whole study, a single batch of freeze-dried isolates was prepared using an Epsilon 2-10D LSC lyophilization instrument (Christ, Osterode am Harz, Germany) with the following procedure. Bacterial enrichments were prepared by inoculating 2 mL LB broth, supplemented with 1 mg/L cefotaxime (CTX), with a single colony of the carbapenem-resistant or ESBL-producing isolates, while cultivation of *E. coli* strain ATCC 25922 was conducted in LB without antimicrobials. The inoculated broths were cultured for 16–20 h at 37 ± 2°C on a rotational shaker (180 rpm). Following incubation, 150 μL of the respective cultures was transferred into 2 mL of fresh LB for further incubation at 37 ± 2°C until an optical density (OD_600 nm_) between 0.5 and 0.6 was reached. Thereafter, the culture was diluted to 1:10,000 (10^3^ to 10^4^ CFU/mL) and centrifuged at 4,000 g for 10 min. The bacterial cell pellet was resuspended in lyophilization medium [glutamic acid 10.0 g/L; NaOH (8%) 30.0 mL/L; Bacto tryptone 25.0 g/L; sucrose 50.0 g/L; thiourea (CH_4_N_2_S) 2.0 g/L] and was applied in aliquots of 1 mL to the borosilicate glasses. Lyophilization was conducted according to the standardized and validated procedure of the German Federal Institute for Risk Assessment (BfR) (freezing; condenser cooling to −50°C; vacuuming the drying chamber; main drying and post-drying). After lyophilization, the bacterial concentrations (CFU/mL) of the freeze-dried bacteria were determined under selective and non-selective conditions. The bacterial concentration after resuspension, plating on LB agar without any supplement and incubation overnight at 37°C accounted between 7.5 and 45 × 10^3^ CFU/mL.

### Enumeration of Bacteria on Different Media

To evaluate the performance of different agar types and the impact of increasing storage time of ChromID^®^ CARBA plates, bacterial growth on different media was determined weekly over a period of 25 weeks as described below. Therefore, the respective lyophilisates of the bacteria were resuspended in 1 mL double-distilled water and incubated for 30–60 min at room temperature for adaption of the bacteria. To exclude potential effects of the commercial agar of the growth performance of the bacteria, five different ChromID^®^ CARBA batches were tested. Aliquots (50 μL) of the lyophilisate suspensions were plated on ChromID^®^ CARBA plates. For comparative analysis of the commercial agar to other media, the same lyophilisate suspensions were also applied to LB agar and to MacConkey agar supplemented with 1 mg/L CTX and 0.125 mg/L meropenem (MEM) (McC+CTX+MEM). Both LB agar and McC+CTX+MEM were produced *in-house* at the BfR. After an incubation period of 16–18 h at 37 ± 2°C, the bacterial concentration (CFU/mL) was determined. For comparability, the storage time the bacterial growth on the five ChromID^®^ CARBA batches was calculated according to the expiry date.

**FIGURE 1:**
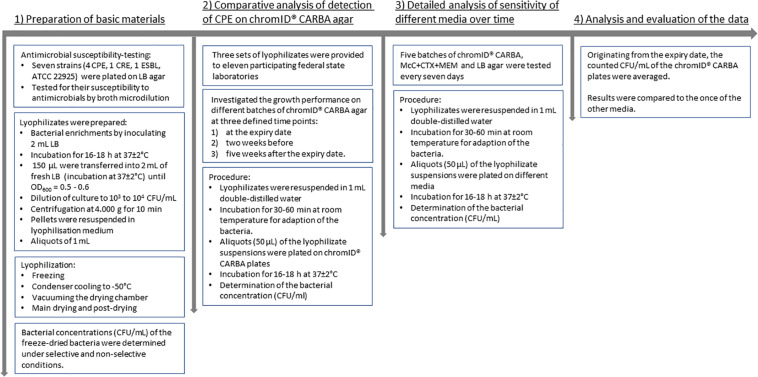
Flow diagram of the systematic procedure.

Freeze-dried bacteria were also provided to 11 participating federal state laboratories for comparative investigation. The laboratories of Baden-Württemberg (Fellbach, Karlsruhe), Bavaria (Oberschleißheim), Hesse (Gießen), Lower Saxony (Cuxhaven, Oldenburg), North Rhine-Westphalia (Arnsberg, Detmold, Krefeld), Saxony (Chemnitz), and Saxony-Anhalt (Stendal) conducted growth investigation according to the specifications of the BfR. Overall, the selectivity of the ChromID^®^ CARBA agar was tested on seven different batches. In contrast to the long-term procedure of the BfR, the federal state laboratories investigated the growth performance of the different isolates on different selective media at three defined time points: (i) at the expiry date, (ii) 2 weeks before, and (iii) 5 weeks after the expiry date. The systematic procedure of all steps is shown in Figure 1.

## Results

Commercial selective medium is not suitable for routine monitoring purposes on isolates from livestock and food that exhibit low carbapenem MIC values.

**FIGURE 2:**
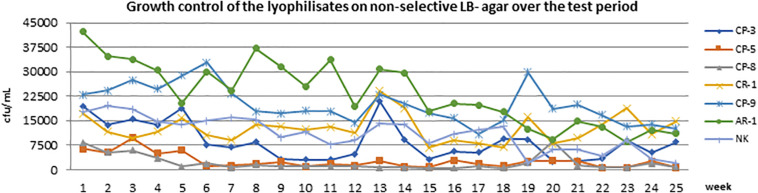
The growth of all lyophilisates on non-selective LB agar over the entire test period of 25 weeks. The *x*-axis (abscissa) shows the time in weeks, and the *y*-axis (ordinate) shows CFU/mL.

Therefore, the commercial ChromID^®^ CARBA agar was tested out of date because of previous observations and experiences. Freeze-dried bacteria, which were produced within this study, showed growth between 5 × 10^2^ CFU/mL and 4.5 × 10^4^ CFU/mL (Figure 2). Over the 25-week period, the bacterial titer of the isolates did not change significantly under non-selective conditions (LB). However, we observed a slight decrease in the bacterial titer for the isolates AR-1, CR-1, and CP-8 over the first time points of enumeration (Figure 2). After 25 weeks, all isolates were still reliably detectable under non-selective conditions (Figure 2).

The results of the comparative analysis are summarized in Supplementary Table S2. Results of comparative testing of the different federal state laboratories were very similar. They indicated that only one isolate (CP-9) could be detected on the used agar batches at all three time points. This respective isolate, a human *E. coli* which carried the gene *bla*_KPC–__2_, exhibited a high MIC values against the tested carbapenems (Table 1). None of the other isolates showed bacterial growth at the selected time points.

**FIGURE 3:**
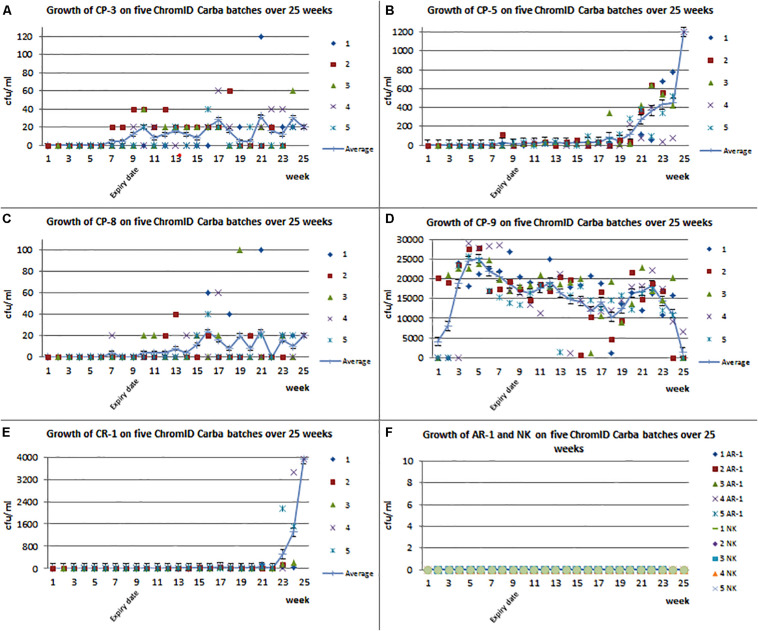
The *x*-axis shows the time in weeks where “9” the expiry date is. The *y*-axis shows CFU/mL. **(A)** Growth of CP-3 on five tested batches of ChromID^®^ CARBA agar. Moreover, the average of all batches is shown. **(B)** Growth of CP-5 on five tested batches of ChromID^®^ CARBA agar. Moreover, the average of all batches is shown. **(C)** Growth of CP-8 on five tested batches of ChromID^®^ CARBA agar. Moreover, the average of all batches is shown. **(D)** Growth of CP-9 on five tested batches of ChromID^®^ CARBA agar. Moreover, the average of all batches is shown. **(E)** Growth of CR-1 on five tested batches of ChromID^®^ CARBA agar. Moreover, the average of all batches is shown. **(F)** Growth of the both control strains NK and AR-1 on five tested batches of ChromID^®^ CARBA agar.

To investigate the suitability of the ChromID^®^ CARBA agar for the detection of CPE exhibiting low carbapenem MIC values, the growth ability of the aforementioned isolates was investigated at the NRL-AR over a time period of 25 weeks using five different batches of the agar. The diagrams 2A–2E show that at the beginning of the test, bacterial growth could only be detected for isolate CP-9, exhibiting high MIC values for the carbapenems ertapenem, imipenem, and meropenem (Table 1 and Supplementary Table S1). Slight bacterial growth could be detected for the isolates CP-3, CP-5, and CP-8 2 weeks before expiry date (week 7) (Figures 3A–C). This detection increased over time and also after the expiry date. The carbapenemase-producing isolate CP-9 showed a highly reliable average growth rate between 5.0 × 10^3^ and 2.5 × 10^4^ CFU/mL at all points of investigations over the 25 weeks (Figure 3D). At the end of the trial, four carbapenem-resistant isolates (CP-3; CP-5; CP-8; and CR-1) showed increased growth rates (average: CP-3 with *bla*_VIM–__1_: 4 to 32 CFU/mL; CP-5 with *bla*_NDM–__1_: 4 to 368 CFU/mL; and CP-8 with *bla*_VIM–__1_: 4 to 24 CFU/mL). The CR-1 isolate that exhibits no carbapenemase-production showed growth rates between 20 and 3920 CFU/mL and was first detected 1 week before the expiry date of the media (Figure 3E). The quality control isolate AR-1 (Table 1 and Supplementary Table S1) and the control strain ATCC 25922 did not grow on ChromID^®^ CARBA plates during the investigated period.

Supplementing 1 mg/L CTX and 0.125 mg/L MEM to the agar basis of the MacConkey medium can overcome growth inhibition during cultivation of low-carbapenem MIC-exhibiting isolates.

The growth performance of the abovementioned isolates was also determined on McC+CTX+MEM agar. In contrast to ChromID^®^ CARBA agar, all carbapenem-resistant isolates were reliably detected at all time points within the 25-week period of investigation (Table 3). The growth of the CP-9 isolate was similar on McC+CTX+MEM, ChromID^®^ CARBA agar as well as under non-selective conditions (LB agar) ranging between 2.4 × 10^3^ and 3.3 × 10^4^ CFU/mL. With the focus on the isolates (AR-1 and NK) used as quality controls, McC+CTX+MEM has a reliable specificity of up to 22 weeks, as within this period no bacterial growth was observed.

**TABLE 3:** Growth of the seven lyophilisates tested once a week over 25 weeks on MacConkey agar supplemented with 0.125 mg/L meropenem and 1 mg/L cefotaxime.

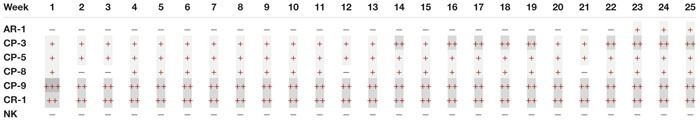

*“−” means no growth; “+” means 1–100 cfu/mL; “++” means 100–1000 cfu/mL; “+++” means ≥1000 cfu/mL.*

## Discussion

Previous experiments in our laboratory showed that the ChromID^®^ CARBA agar becomes more sensitive with increasing storage time. Based on the observations and experiences within the laboratory routine (International Organization for Standardization, 2006; Fischer et al., 2013a, b, 2017; Irrgang et al., 2017, 2019), detection of carbapenemase-producing bacteria is challenging using the EURL-AR method in combination with ChromID^®^ CARBA agar. We assume that the concentration of different ingredients and thus the selectivity will decrease with time or after the expiry date. This justifies the choice of times to determine a possible decrease in antimicrobial concentration after the shelf life of ChromID^®^ CARBA agar.

Within this study, we observed strong differences in the growth performance of the used carbapenem-resistant isolates on the selective media. These different growth performances exceed the observed slight decrease of AR-1, CR-1, and CP-8 during the first time points of enumeration, which seems to represent a natural adaption of the specific isolates to the prevailing cultivation conditions (Figure 2).

As previously reported (Fischer et al., 2012; Köck et al., 2018), carbapenem-resistant isolates from food products or livestock exhibit significantly lower MIC values for the respective carbapenems than clinical isolates. There may be different reasons for this observation. It could be due to molecular conditions, such as a variable number of gene copies, a different expression of the genes, or a different genetic localization (plasmid versus chromosomal DNA). It could also be due to the different antimicrobial selection pressures to which the strains are exposed. As carbapenems are not licensed for use in food animals, previous exposure of the strains with a livestock origin is unlikely. The chosen isolates for the evaluation of the selective cultivation step of the detection procedure represent different bacterial genera and carbapenem resistance genes as well as resistance mechanisms (Viau et al., 2012; Köck et al., 2018). The *E. coli* isolate CP-9 carries a *bla*_KPC–__2_ gene and exhibited high MIC values against all tested carbapenems. CP-9 represents typical MIC values for carbapenems of human isolates (Supplementary Table S1). Other target isolates, except CP-5 (the NDM-producing *Salmonella*) exhibited carbapenem resistance but showed only MICs ranging around the current cutoff values (Supplementary Table S1). As the used isolates exhibited very low MIC values, we suppose that the remaining concentration of the supplemented antimicrobials is still high enough to prevent a growth of these isolates.

The KPC enzymes belong to Amber class A carbapenemases and use a serine-based hydrolytic mechanism to inactivate carbapenems. In contrast, the VIM-1 and NDM-1 enzymes belong to Ambler class B carbapenemases and convey the resistances by a zinc-catalyzing active site (Codjoe and Donkor, 2017). Viau et al. (2016) reported that most of their ten tested media performed reasonably well with different screening methods for the detection of class A enzymes. Performance with class B and D enzymes was more variable. However, Viau and co-workers used up to 180 different CRE originating from human clinical sources. They determined a good performance of the ChromID^®^ CARBA medium if tested with rectal/perirectal swabs (Jeon et al., 2015). The differences between CPE from human samples and CPE from non-clinical animal samples have not been investigated systematically so far as the number of available non-clinical animal isolates is still limited. As the ingredients of the ChromID^®^ CARBA agar are unknown and have not been provided by the manufacturers on request, it is difficult to assess the selective additives of the medium and to expect their influence on the cultivation of CPE with low MIC. It might be that an additive supports the serine-based hydrolytic resistance mechanism in a better way than the zinc-based mechanism.

Within our study, CP-9 was the only isolate that was consistently detected in all participating laboratories and with all selective media. The determined amount of the adjusted, freeze-dried bacteria ranged between 5 × 10^2^ and 4.5 × 10^4^ CFU/mL between the laboratories. The other carbapenem-resistant isolates (CP-3; CP-5; CP-8; and CR-1) showed no detectable growth on the ChromID^®^ CARBA plates within the different federal state laboratories up to 5 weeks after expiration date of the plates (Supplementary Table S2). At this time point, the agar will not be used by accredited laboratories as the agar will be handled as expired according to the recommendations of the manufacturers. However, each of these isolates was reliably detected on McC+CTX+MEM (Table 3). The quality control isolate AR-1, a CTX-M-1-producing *E. coli* with a low phenotypical resistance against ertapenem (Table 1 and Supplementary Table S1), and the quality control strain ATCC 25922 did not grow on ChromID^®^ CARBA plates at any time (Figure 3F). As they grew at the last three time points on McC+CTX+MEM, it can be assumed that the specificity of the plates did not decrease considerably within a period of 20 weeks after production (Table 3). In contrast, the number of CFU of the isolates CP-3; CP-5; CP-8; and CR-1 increased at the end of the investigated time interval. This indicates that the sensitivity of the commercial agar increased over time.

In contrast to our study, other groups found that ChromID^®^ CARBA agar was one of the most sensitive and specific chromogenic medium for the detection of CPE (Vrioni et al., 2012; Papadimitriou-Olivgeris et al., 2014; Simner et al., 2015; Viau et al., 2016). However, in these studies, the MIC values of the CPE are in general much higher than the MIC values of the isolates tested here. In comparison, Vrioni et al. (2012) used clinical isolates with very high MIC values of up to over 32 mg/L to MEM, but they also used some strains with MIC values below the resistance breakpoint. The detection of the isolates with these high MIC values might correlate much more with the detection of CP-9 and is not comparable with the detection of isolates exhibiting low MICs (CP-3, CP-8, and CR-1).

Limited detection of CPE might be also attributable to a loss of the resistance-harboring plasmids during lyophilization (Wagman and Weneck, 1963; Berman et al., 1968; Wein et al., 2019). We can exclude this as an important factor in our study because we observed similar bacterial titers on selective and non-selective *in-house* media (data not shown). We had also confirmed the presence of the plasmid in some representative colonies from selective and non-selective media.

Overall, we have detected more CFU/mL by using the conventional MacConkey agar supplemented with antibiotics over the entire period for almost all isolates of this study. Due to the lacking resistance against carbapenems, the quality control strain ATCC 25922 could not be detected on McC+CTX+MEM, indicating the appropriate specificity of the medium. Some spontaneous growth of the *E. coli* isolate AR_1 was observed 22, 23, and 24 weeks after agar production, indicating that the prepared agar worked with a high specificity for up to 22 weeks. In contrast to this observation, the AR_1 isolate could not be detected on the ChromID^®^ CARBA agar. The concentration of meropenem (0.125 mg/L) in the supplemented agar is lower than the current ECOFFs (0.25 mg/L) (Giske et al., 2013). Based on this concentration, CPE with low MIC values are also able to grow successfully and will further be detected within the monitoring programs. Viau et al. (2012) reported about “silent” dissemination of *K. pneumoniae*, because some isolates were not identified as carbapenem-resistant based on their MIC, even though they carried a carbapenemase gene and showed slightly increased MIC values against ertapenem and meropenem (Fischer et al., 2012).

Berthoin et al. (2010) reported that the stability of meropenem in solutions depends on time, temperature, and concentration (Berthoin et al., 2010). These observations were supported by Keel et al. (2011). A potential effect of light on the stability of meropenem can be excluded, as the agar plates were only exposed to light during plating and visual inspection. Therefore, it was assumed that the stability of meropenem in the media might be limited. In our study, the McC+CTX+MEM agar was stably usable for up to 22 weeks without a loss of specificity when stored at 4 ± 2°C. The supplementation with cefotaxime supported the selective effect, but for OXA-48 producers this antibiotic should be omitted. Changes in sensitivity and specificity of MacConkey agar supplemented with meropenem only over time were not tested in our study. Adler et al. (2011) used MacConkey agar supplemented with imipenem at 1 mg/mL (McC+IMI) and compared this medium with a commercial selective medium (CHROMagar KPC, CHROMagar Company, Paris, France). They described a similar performance of both media during clinical evaluation. Moreover, they argued with the lower costs of McC+IMI to prefer it for detection of CRE in their study (Adler et al., 2011). We decided to use meropenem as supplement because it is more stable than imipenem (Zhanel et al., 2007). Based on our data, we recommend using McC+CTX+MEM for selective cultivation of carbapenem-resistant isolates from food products and livestock samples.

Another important difference between the different agars is that the ingredients of the *in-house* prepared agar are known. In McC+CTX+MEM agar, no further substances were supplemented for inhibition of accompanying bacteria. It is possible that the commercial selective medium comprises unspecified substances (e.g., zinc that is used to inhibit non-carbapenemase-producing bacteria), which may also suppress the growth of some CPE, e.g., with a low MIC. Although less selective substances are used, no increased difficulties were found with the accompanying flora in other experiments so far.

A limitation of this study is that only one batch of McC+CTX+MEM plates was used throughout the experiment. This was due to its function as a control medium, and it was not the primary aim to compare both media.

## Conclusion

The results of our study indicate that the ChromID^®^ CARBA agar is not suitable for the cultural detection of CPE with low MIC to carbapenems, while clinical isolates with higher MIC values can be detected reliably. Overall, the use of the ChromID^®^ CARBA agar in monitoring programs may lead to an underestimation of the CPE occurrence in livestock and food as the method lacks sensitivity.

We further provide data, that MacConkey agar with 1 mg/L cefotaxime and 0.125 mg/L meropenem provides a better sensitivity for the detection of such isolates and may be more suitable for the carbapenemase monitoring in livestock and food.

## Data Availability Statement

All datasets presented in this study are included in the article/Supplementary Material.

## Ethics Statement

This study uses strains obtained from humans (*E. coli* CP-9), livestock (*E. coli* AR-1 and CP-3, *Klebsiella pneumoniae* CR-1), wildlife (*Salmonella* Corvallis CP-5) and seafood (*Vibrio parahaemolyticus* CP-8). All isolates were part of the strain collection of the German NRL AR and had been submitted to the NRL in the framework of its routine tasks in monitoring and surveillance programs or during previous scientific collaborations. None of the isolates was collected specifically for the current study. Therefore we did not consider it necessary to seek for ethical approval for using the isolates.

## Author Contributions

JH, NP, and AI designed the study and developed the draft of the manuscript. NP performed the preparation of adjusted, freeze-dried bacteria, and the determination of the growth performance of the isolates at the NRL-AR. MG, AI, and JH initially characterized the carbapenem-resistant isolates. SB, JF, SH, HL, UM, MM, SR, AS, SS, BS, and PZ performed the individual growth investigations on the commercial agar at the different federal state laboratories. NP, AI, JH, MG, AK, B-AT, SS, and DM performed the data analysis. All authors supported the finalization of the manuscript and supported editing of the final version.

## Conflict of Interest

The authors declare that the research was conducted in the absence of any commercial or financial relationships that could be construed as a potential conflict of interest.

## Footnotes

1 https://www.eurl-ar.eu/protocols.aspx

2 www.eucast.org
