# Supplementary material for: ChromID® CARBA Agar Fails to Detect Carbapenem-Resistant Enterobacteriaceae With Slightly Reduced Susceptibility to Carbapenems
Source: Front Microbiol. 2020 Aug 11;11:1678. doi: 10.3389/fmicb.2020.01678 (PMC7432429; doi:10.3389/fmicb.2020.01678)
Supplement: Supplementary file 1 [file Data_Sheet_1.PDF]

Isolate	AMP	AZI	FEP	CHL	CIP	COL	ERP	FOT	FOX	GEN	IMI	MERO	NAL	SMX	TAZ	TET	TGC	TMP
CP-3	>64	8	16	64	0.03	≤1	0.12	>64	32	1	4	1	≤4	32	64	4	0.5	>32
	>64	8	16	128	≤0.015	≤1	0.12	>64	16	2	4	1	≤4	32	64	≤2	0.5	>32
	>64	8	16	128	≤0.015	≤1	0.12	>64	32	1	4	1	≤4	16	64	4	0.5	>32
CP-5	>64	64	16	>128	0.5	4	>2	>64	>64	1	4	8	32	>1024	>64	64	0.5	≤0.25
	>64	32	32	>128	0.5	2	>2	>64	>64	1	4	8	16	>1024	>64	64	0.5	≤0.25
	>64	32	32	>128	0.5	2	>2	>64	>64	2	4	8	16	>1024	>64	64	0.5	≤0.25
CP-8	>64	≤2	32	≤8	0.12	8	0.25	>64	>64	1	2	0.25	≤4	≤8	>128	≤2	≤0.25	2
	>64	≤2	32	≤8	0.06	8	0.25	>64	>64	1	1	0.25	≤4	≤8	>128	≤2	≤0.25	2
	>64	≤2	32	≤8	0.12	8	0.5	>64	>64	1	4	0.25	≤4	≤8	>128	≤2	≤0.25	2
CP-9	>64	8	32	>128	>8	≤1	>2	>64	>64	>32	4	8	>128	≤8	32	>64	≤0.25	≤0.25
	>64	8	32	>128	>8	≤1	>2	>64	>64	>32	4	8	>128	≤8	64	>64	≤0.25	≤0.25
	>64	8	32	>128	>8	≤1	>2	>64	>64	>32	4	8	>128	≤8	32	>64	≤0.25	≤0.25
CR-1	>64	16	>32	128	>8	≤1	2	>64	64	≤0.5	0.5	0.5	>128	64	>128	4	0.5	>32
	>64	16	>32	128	>8	≤1	>2	>64	64	≤0.5	0.5	0.5	128	64	>128	4	0.5	>32
	>64	16	>32	128	>8	2	2	>64	64	≤0.5	1	0.5	>128	128	>128	8	0.5	>32
AR-1	>64	8	>32	>128	>8	16	0.25	>64	16	>32	0.5	0.6	>128	>1024	4	>64	≤0.25	>32
	>64	8	>32	>128	>8	16	0.25	>64	16	>32	0.5	0.6	>128	>1024	4	>64	≤0.25	>32
	>64	8	>32	>128	>8	16	0.25	>64	16	32	0.5	0.6	>128	>1024	4	>64	≤0.25	>32
NK	4	4	≤0,06	≤8	≤0.015	≤1	≤0.015	≤0.25	2	1	0.25	≤0.03	≤4	≤8	≤0.5	≤2	≤0.25	≤0.25
	8	4	≤0,06	≤8	≤0.015	≤1	≤0.015	≤0.25	2	1	0.25	≤0.03	≤4	≤8	≤0.5	≤2	≤0.25	≤0.25
	4	4	≤0,06	≤8	≤0.015	≤1	≤0.015	≤0.25	4	1	0.25	≤0.03	≤4	≤8	≤0.5	≤2	≤0.25	≤0.25

Supplemental Material Table S1: Detailed information of the threefold determined minimal inhibitory concentration (MIC) values of the isolates. MIC values are given in mg/L. The interpretation of the MIC values in wildtype (susceptible) and non-wildtype (resistant) was indicated in green and red, respectively. Ampicillin (AMP); Azithromycin (AZI); Cefepime (FEP); Chloramphenicol (CHL); Ciprofloxacin (CIP); Colistin (COL); Ertapenem (ERP); Cefotaxime (FOT); Cefoxitin (FOX); Gentamicin (GEN); Imipenem (IMI); Meropenem (MERO); Nalidixic acid (NAL); Sulfamethoxazole (SMX); Ceftazidime (TAZ); Temocillin (TEM); Tetracycline (TET); Tigecycline (TGC); Trimethophrim (TMP)

Laboratory	1			2			3			4			5			6			7			8			9			10			11		
Batch number	1007341350			1007322070			1007408770			1007436550			1007401150			1007431050			1007401150			1007401150			1007382940			1007431050			1007424160		
Time point	+2	0	-5	+2	0	-5	+2	0	-5	+2	0	-5	+2	0	-5	+2	0	-5	+2	0	-5	+2	0	-5	+2	0	-5	+2	0	-5	+2	0	-5
AR-1	-	-	-	-	-	-	-	-	-	-	-	-	-	-	-	-	-	-	-	-	-	-	-	-	-	-	-	-	-	-	-	-	-
CP-3	-	-	-	-	-	-	-*	-	-	-	-	-	-	-	-	-	-	-	-	-	-	-	-	-	-*	-	-	-	-	-	-	-	-
CP-5	-	-	-	-	-	-	-	-	-	-	-	-	-	-	-	-	-	-	-	-	-	-	-	-	-*	-	-*	-	-	-	-	-	-
CP-8	-	-	-	-*	-*	-	-*	-*	-	-*	-	-	-	-	-	-	-	-	-	-	-	-	-	-	-	-	-	-	-	-	-	-	-
CP-9	++	++	++	+++	+++	+++	++*	++	+++	++	++	++	+++	++	+++	++	+++	+++	+++	++	+++	+++	+++	+++	+++	++	++	+++	++	++	+++	+++	+++
CR-1	-	-	-	-	-	-	-	-	-	-	-	-	-	-	-	-	-	-	-	-	-	-	-	-	-	-	-	-	-	-	-	-	-
NK	-	-	-	-	-	-	-	-	-	-	-	-	-	-	-	-	-	-	-	-	-	-	-	-	-	-	-	-	-	-	-	-	-

Supplemental Material Table S2: Growth of the seven lyophilisates on three timepoints (0=the expiry date; +2=two weeks before; -5=five weeks after) tested by eleven laboratories on seven different batches of ChromID Carba agar. “-“ means no growth; “+” means 1 to 100 cfu/ml; “++” means 100 to 1000 CFU/ml; “+++” means ≥1000 CFU/ml. * marks the invalid result of lyophilisates on non-selective agar.
